# Maternal iron deficiency, preterm birth, and disrupted microbial inheritance: a proposed double-hit hypothesis

**DOI:** 10.3389/fcimb.2026.1953925

**Published:** 2026-10-23

**Authors:** Fouzia Zahid Ali Khan, Syed Arham Ali

**Affiliations:** 1 Department of Obstetrics and Gynecology, Ali Hospital, Islamabad, Pakistan; 2 Rawal Institute of Medical Sciences, Islamabad, Pakistan

**Keywords:** gut-vaginal axis, infant microbiome, maternal iron deficiency, maternal microbial transmission, mother-to-infant transmission, preterm (birth), vaginal dysbiosis

## Abstract

The maternal microbiome is the infant’s primary microbial inheritance, transmitted mainly through the maternal gut, vaginal delivery, breast milk, and skin-to-skin contact. Maternal iron deficiency (ID), with anemia, is a common and modifiable nutritional condition associated with impaired immune function and increased preterm birth (PTB) risk, whereas evidence for ID without anemia remains limited. It may influence maternal microbial ecology; however, its potential effect on maternal-to-infant microbial transmission remains unexplored. This review, conducted across PubMed, Embase, and Web of Science (January 2000–June 2026), proposes a double-hit hypothesis defined by the source and biological context of disruption rather than by a strictly temporal sequence. The first hit comprises potentially altered maternal-origin microbial and immunologic inputs from the vaginal, gut, and breast milk ecosystems; the second hit comprises PTB and neonatal intensive care (NICU)-associated disruption of early microbial succession. Human evidence directly linking ID to vaginal dysbiosis is limited to a single study, evidence relating maternal iron status to milk lactoferrin is inconsistent, and the contribution of vaginal communities to persistent infant gut colonization remains uncertain whereas vaginal exposure may have transient ecological or immunologic effects. No study has simultaneously evaluated maternal iron biomarkers, vaginal community state type (CST), breast milk composition, maternal gut microbiome and longitudinal infant gut microbiota. The proposed framework is therefore hypothesis-generating and identifies testable pathways through which maternal ID may alter maternal microbial and immunologic inputs, while also being associated with an increased risk of PTB. PTB, together with the resulting prematurity-related vulnerability and subsequent postnatal exposures, may further disrupt early infant microbiome development.

## 1 Introduction

The first 1, 000 days of life, from conception to age 2, represent a critical window for microbiome-mediated immune and metabolic programming (Ferretti et al., 2018; Bogaert et al., 2023; Suárez-Martínez et al., 2023; Bautista and López-Cortés, 2025; Flores Ventura et al., 2025). Maternal microbial communities constitute major sources of the infant’s early microbial inheritance. Microbial inheritance is used here as an operational concept encompassing the acquisition of maternal microorganisms, microbial products, and related functional inputs during delivery and early postnatal life. It spans a gradient from transient inoculation, such as vaginal and skin inoculum, to durable selective strain-level colonization, principally from the maternal gut and secondarily from breast milk. The concept includes both the composition of the transferred microbiota and the functional capacity these organisms confer to the developing infant, particularly in terms of immune programming and metabolic development (Wang et al., 2020; Du et al., 2025).

Multiple large-scale studies have demonstrated substantial contributions of maternal source communities to early infant microbiota composition across six maternal body sites, including vaginal, fecal, skin, nasal, oral, and breast milk niches, though vaginal and skin contributions are transient (Ferretti et al., 2018; Bogaert et al., 2023).

Maternal microbial inheritance is biologically consequential, as microorganisms and microbial products transferred at birth and during lactation actively shape infant immune maturation, gut metabolic capacity, and susceptibility to inflammatory and atopic disease throughout childhood (Davis et al., 2022; Bogaert et al., 2023). When this inheritance is disrupted due to underlying maternal medical conditions, cesarean delivery (CD), antibiotic exposure, or maternal microbial dysbiosis, such disruptions extend far beyond the neonatal period and can have lifelong consequences (Robertson et al., 2019; Low et al., 2025).

Maternal nutrition is one potential determinant of microbial and immunological development, and several studies have examined the association involving various macro- and micronutrients like vitamins A, C, D and E (Christian et al., 2011; Tuddenham et al., 2019; Khaikin et al., 2025; Yang and Gong, 2026). Iron deficiency is the most prevalent micronutrient deficiency worldwide, affecting roughly 2 billion people across the globe. It disproportionately impacts several vulnerable demographic groups including 30% of premenopausal and 37% of pregnant women (Kirthan and Somannavar, 2024; Frost and Drakesmith, 2025). Iron is an essential cofactor for the NADPH oxidase enzyme complex responsible for neutrophil oxidative burst which is the principal mechanism by which neutrophils kill invading bacteria (Frost and Drakesmith, 2025; Frost et al., 2022). Yet whether maternal iron status influences vaginal microbial ecology and thereby shapes infant microbiome development through altered maternal microbial inputs remains largely unexplored.

The central thesis of this paper is that maternal ID may create a *double-hit* on the infant’s microbial inheritance. The first hit comprises potentially altered maternal microbial and immunologic inputs arising through interconnected vaginal, gut-vaginal, and breast milk pathways. The second hit occurs if the pregnancy ends in PTB, an outcome associated with maternal ID, and the infant is exposed to microbial disruption related to prematurity and the NICU environment. Early delivery may itself curtail the late-gestation window of maternal-to-infant immune priming, most notably third-trimester transplacental antibody transfer, compounding this disruption (Marchant et al., 2017). Among infants born preterm, subsequent exposure to broad-spectrum antibiotics, delayed or altered enteral feeding, invasive care, and nosocomial organisms may further perturb early microbial succession, documented by multiple studies (Shao et al., 2019; Reyman et al., 2022; Kiu et al., 2025; Tao et al., 2026).

This review synthesizes evidence across maternal ID, vaginal microbiome ecology, maternal-to-infant microbial transmission, breast milk immunology, and preterm neonatal microbiome development. We use this evidence to map the biological continuum through which maternal ID may shape infant microbiome development and to identify where direct evidence is limited, conflicting, or absent. The double-hit model is presented as a hypothesis-generating framework rather than an established causal pathway, and each proposed connection requires direct empirical testing.

## 2 Methods

A narrative review of peer-reviewed literature published between January 2000 and June 2026 was conducted across PubMed, Embase, and Web of Science, supplemented by manual reference tracking. Search terms were combined using Boolean operators (AND, OR) and included: “maternal anemia,” “iron deficiency,” “pregnancy,” “vaginal microbiome,” “bacterial vaginosis,” “*Lactobacillus*,” “community state type,” “maternal-to-infant microbiome transfer,” “vertical transmission microbiome,” “neonatal gut microbiome,” “breast milk microbiome,” “lactoferrin breast milk,” “human milk oligosaccharides,” “vaginal seeding,” and “preterm infant gut microbiome”. Reporting was guided in principle by the Scale for the Assessment of Narrative Review Articles (SANRA) to improve transparency and reproducibility of the literature search, while explicitly not claiming systematic-review status. Human studies were prioritized, while experimental animal and *in vitro* studies were included when relevant to biological plausibility. Studies were selected according to their relevance to the individual components of the framework rather than through exhaustive systematic-review screening. Non-English publications were excluded because translation resources were unavailable. Conference and grey literature were excluded because they frequently lacked sufficient methodological detail, complete outcome reporting, or peer-reviewed data to permit critical evaluation of the proposed hypothesis. These exclusions may have introduced language and publication bias and may have resulted in the omission of relevant evidence.

This approach is inherently hypothesis-generating and cannot substitute for systematic evidence synthesis. Where evidence is limited, conflicting, or absent, this is stated explicitly. A summary of key studies supporting each route of the hypothesis is provided in **Table 1**.

**Table 1:** Evidence informing the proposed double-hit framework linking maternal iron deficiency, maternal-origin microbial inputs, preterm birth, and infant microbiome development.

Framework component	Study	Design and population	Principal finding	Relevance to the proposed framework	Evidence type/directness	Key limitation
Foundational: Maternal–infant transmission	Ferretti et al. (2018)	Longitudinal strain-level metagenomic study of mother–infant pairs; multiple maternal body sites	Maternal gut strains were more persistent and ecologically adapted in the infant gut; maternal vaginal and skin strains were mainly transient.	Supports distinguishing transient exposure from persistent infant-gut colonization.	Direct human, strain-level evidence	Population and sampling intervals limit generalizability; persistence does not establish clinical effects.
Foundational: Maternal source communities	Bogaert et al. (2023)	Prospective cohort; 120 mother–infant pairs; six maternal and four infant niches during the first month	An estimated 58.5% of infant microbiota composition across sampled niches was attributable to maternal source communities; contributions differed by niche and delivery mode.	Supports multiple, partly compensatory maternal routes rather than a single dominant route.	Direct human, multi-niche cohort evidence	The 58.5% estimate applies across sampled infant niches and should not be interpreted as the infant gut alone.
Foundational: Limited vaginal contribution	Dos Santos et al. (2023)	Canadian prospective cohorts; cpn60 profiling, molecular strain typing, culture and whole-genome sequencing	Maternal vaginal community composition did not predict early infant stool composition; near-identical vaginal–stool strains were detected irrespective of delivery mode.	Challenges birth-canal exposure as the sole explanation for persistent infant-gut strain sharing.	Direct human observational evidence that limits the proposed pathway	Detection of shared strains does not establish their anatomical source or direction of transmission.
Foundational: Gut versus vaginal source	Xie et al. (2025)	Pregnancy–neonatal cohort; maternal gut and vaginal samples with neonatal meconium analysis	Maternal gut microbiota showed a greater contribution to meconium than maternal vaginal microbiota among vaginally delivered infants.	Supports a greater role for maternal gut-derived communities in persistent infant-gut colonization.	Indirect-to-direct human observational evidence	Meconium-based inference is sensitive to low biomass, contamination, and source-tracking assumptions.
Foundational: Gut versus vaginal source	Ronde et al. (2025)	Mother–infant cohort; maternal rectal/vaginal and neonatal gut amplicon sequence variants	By 28 days, variants shared with maternal rectal microbiota represented a greater proportion of the neonatal gut than variants shared with vaginal microbiota.	Supports persistence of gut-associated over vaginal-associated microbial signals.	Human observational evidence	Amplicon sequence sharing cannot definitively establish strain-level vertical transmission.
Foundational: Vaginal *Lactobacillus* and gut-vaginal sharing	Oguri et al. (2025)	Japanese maternal-neonatal cohort; vaginal, gut, and neonatal gut sampling	Higher vaginal *Lactobacillus* abundance in early pregnancy was associated with favorable gestational outcomes; bacteria shared between maternal gut and vaginal microbiome became dominant in the neonatal gut over time	Supports both the vaginal route’s clinical relevance and a gut-vaginal-to-infant transmission link within the same cohort.	Human observational cohort evidence	Single population; does not establish maternal iron status as a determinant of the observed vaginal or shared taxa
Foundational: CST and infant gut composition	Bhattacharyya et al. (2023)	Small observational study; infants of mothers classified as CST III vs. CST IV (n=25)	Infant gut microbiome composition differed by maternal CST classification at delivery.	Provides preliminary support that maternal CST specifically, not just delivery mode, may shape infant gut composition.	Human Observational evidence	Sample size (n=25) is too small for definitive conclusions; unreplicated.
Foundational: *L. crispatus* in meconium	Simonyté Sjödin et al. (2025)	Larger cohort; 264 mothers and 266 infants; meconium sampling within 48 hours of birth	*L. crispatus* was detected in meconium of vaginally delivered infants within 48 hours of birth.	Supports early vaginal-organism detection in the infant gut immediately after birth.	Human observational evidence, larger cohort	16S rRNA resolution could not establish strain-level transmission from mother to infant
First hit: Vaginal route anchor	Verstraelen et al. (2005)	Nested case–control study in early pregnancy; 18 women with vaginosis-like microflora and 80 controls	Subclinical iron deficiency was independently associated with vaginosis-like microflora (adjusted OR 4.5; 95% CI 1.4–14.2).	Provides the only direct human epidemiological link between maternal iron status and vaginal dysbiosis in the framework.	Human observational study (single, unreplicated & small)	Small case group; modified Nugent assessment; no sequencing, longitudinal CST assessment or infant outcomes.
First hit: Host-defense mechanism	Frost and Drakesmith (2025); Frost et al. (2022)	Review and experimental studies of iron availability and neutrophil biology	Iron availability influences neutrophil production and oxidative antimicrobial function.	Provides a possible host-defense mechanism through which ID could affect mucosal ecology.	Indirect mechanistic evidence	Not demonstrated specifically in the pregnant cervicovaginal environment or linked to CST transitions.
First hit: Lactobacillus iron biology	Elli et al. (2000); Bosma et al. (2021)	*In-vitro* and comparative metabolic studies	*Lactobacilli* have relatively low iron requirements and may use manganese-dependent pathways in place of iron-requiring functions.	Explains why systemic iron restriction is unlikely to affect lactobacilli through simple iron starvation.	*In-vitro*/comparative mechanistic evidence	Species- and strain-specific findings; clinical relevance to pregnancy and vaginal CSTs is uncertain.
First hit: Local iron and Lactobacillus function	Martín et al. (2015)	*In-vitro* vaginal epithelial adhesion and functional experiments	Experimental Fe^3+^ exposure enhanced selected *L. jensenii* adhesion and functional properties.	Suggests that local iron availability could affect Lactobacillus function independently of systemic iron stores.	*In-vitro* mechanistic evidence	Does not demonstrate that systemic maternal ID reduces local vaginal iron or Lactobacillus fitness *in vivo*.
First hit: CSTs and PTB	McKee et al. (2025; ECHO)	Pregnancy cohort examining vaginal community state types and PTB	*L. iners*-dominant and diverse low-L*actobacillus* communities were associated with different PTB risks relative to *L. crispatus*-dominant communities.	Supports clinical relevance of vaginal community structure within the proposed pathway.	Human observational evidence	Association does not establish that maternal ID caused the CST or that CST mediated an ID–PTB relationship.
First hit: Species-specific immune interaction	Decout et al. (2024)	Experimental immunology study of vaginal bacterial surface-layer proteins and innate immune receptors	*L. crispatus* surface-layer proteins showed selective interaction with DC-SIGN; *L. iners* and *Gardnerella* exhibited different receptor-interaction profiles.	Provides a possible mechanism for species-specific mucosal immune signaling.	Experimental mechanistic evidence	Does not demonstrate immune programming of human infants following maternal vaginal exposure.
First hit: ID and maternal gut ecology	Dostal et al. (2012); Soriano-Lerma et al. (2022); Coe et al. (2021)	Animal and experimental models of iron deficiency and repletion	Experimental ID altered gut microbial composition and reduced butyrate production (Dostal et al., 2012; Soriano-Lerma et al., 2022); in a mouse model, low-iron challenge caused durable shifts in diversity, with *Prevotellaceae* and *Porphyromonadaceae* taxa failing to recover after iron repletion (Coe et al., 2021)	Provides biological plausibility for an ID-associated maternal gut pathway.	Preclinical mechanistic evidence	Predominantly nonpregnant/animal evidence; taxa and metabolic responses may not translate to pregnant humans.
First hit: Proposed gut–vaginal interaction	El Aila et al. (2009); Fudaba et al. (2021); Kim et al. (2026)	Genomic comparison, observational study in pregnant women and experimental model	Genomic overlap and rectal reservoirs support possible microbial exchange between gut and vaginal communities; experimental work suggests cross-site ecological effects.	Supports a plausible gut–vaginal route involving shared reservoirs and immune/metabolic signaling.	Indirect human and preclinical evidence	Directionality is unresolved; evidence does not establish gut-to-vagina translocation caused by maternal ID.
First hit: Maternal anemia and maternal/infant gut microbiota	Mandell et al. (2025)	Longitudinal Pune cohort; 218 women enrolled at 13–34 weeks and followed 6 months postpartum; infant sampling at 6 months	Maternal anemia (Hb <110 g/L) was associated with differential abundance of selected maternal and infant gut taxa, including *Flavonifractor, Enterococcus, Coprococcus_2*, and *Desulfovibrio.*	Provides indirect human evidence that maternal anemia is associated with maternal and infant gut microbial profiles.	Indirect human longitudinal evidence	Anemia was defined by hemoglobin rather than iron biomarkers; vaginal and milk microbiomes were not assessed, so the transmission route is unknown.
First hit: Breast milk lactoferrin biology	Mastromarino et al. (2014)	Neonatal observational cohort with maternal milk and infant fecal measurements	Milk lactoferrin concentration was associated with neonatal fecal lactoferrin and early fecal *Bifidobacterium* and *Lactobacillus* abundance.	Supports lactoferrin as a possible postnatal microbial-selection mechanism.	Indirect human observational evidence	Does not evaluate maternal ID; associations do not establish that lactoferrin caused colonization differences.
First hit: Maternal iron status and milk lactoferrin	Shashiraj et al. (2006); Fujita et al. (2022); Zhou et al. (2025)	Prospective maternal–milk cohorts across different populations and lactation stages	Most analyses found no association between maternal iron biomarkers or IDA and milk lactoferrin; one cohort reported an association between prenatal MCV and longitudinal lactoferrin trajectories.	Indicates that any maternal iron–lactoferrin pathway is uncertain and may vary by population or lactation stage.	Conflicting/limited human evidence	MCV is an indirect, nonspecific indicator of iron status; studies used heterogeneous biomarkers and sampling schedules.
First hit: Breast-Milk HMOS and infant gut	Ovaska et al. (2026); Kiely et al. (2023); Melki and Litvak (2024)	Finnish population-based cohort (n=517) with infant gut microbiota profiling; supported by molecular studies of HMO utilization	HMOs showed age-dependent, structure-specific associations with infant gut microbiota, and maternal secretor status influenced gut microbiota beta diversity; HMOs selectively support Bifidobacterium (notably B. infantis) via dedicated utilization systems	Supports HMOs as a maternal-origin postnatal factor shaping infant gut colonization, forming part of the breast-milk arm of the first hit	Direct human observational evidence with supporting mechanistic data	No study links maternal iron status to HMO composition; the ID–HMO connection is unexamined and remains a hypothesized gap
First hit: Breast-milk microbial contribution	Pannaraj et al. (2017)	Prospective longitudinal study; 107 healthy mother–infant pairs; 16S rRNA source tracking	Among predominantly breastfed infants, a mean 27.7% of gut bacteria during the first 30 days was attributed to breast milk and 10.3% to areolar skin.	Supports breast milk as an important maternal-origin postnatal input.	Direct human observational evidence based on source tracking	16S source tracking cannot prove strain-level transfer; estimate applied to infants obtaining ≥75% of daily intake from breast milk.
Second hit: Preterm infant dysbiosis	Shao et al. (2019); Tao et al. (2026)	Large neonatal cohort and evidence synthesis of preterm gut development	Preterm infants showed altered succession, reduced Bifidobacterium and enrichment of hospital-associated/opportunistic taxa.	Supports prematurity-associated ecological vulnerability within the second hit.	Consistent human cohort evidence	Gestational age, delivery mode, antibiotics, feeding, and illness severity are difficult to disentangle.
Second hit: NICU-associated disruptors	Zwittink et al. (2018); Reyman et al. (2022); Thänert et al. (2024)	Neonatal cohorts and interventional/longitudinal studies of antibiotics, feeding and hospital-associated organisms	Antibiotic exposure, altered feeding, and acquisition of hospital-associated organisms were associated with disrupted microbial succession.	Supports the NICU-associated component of the second hit.	Human longitudinal/interventional evidence	Exposures cluster with prematurity and illness severity; strength and direction vary by exposure and study.
Second hit: Gut origin of late-onset sepsis	Graspeuntner et al. (2019); Lee et al. (2021)	Prospective neonatal cohorts with longitudinal stool sampling and pathogen comparison	Altered gut profiles preceded late-onset sepsis, and a subset of bloodstream pathogens was strain-matched to earlier gut isolates.	Supports temporal and strain-level links between gut colonization and some cases of neonatal sepsis.	Prospective human temporal/strain-level evidence	Not all sepsis pathogens originated from the gut; preceding dysbiosis does not alone prove causation.
Second hit: ID/IDA and PTB	Benson et al. (2022); Lawani et al. (2026)	Meta-analytic and cohort evidence on maternal iron status and PTB	Maternal IDA was associated with an approximately 1.5-fold increase in PTB odds (OR 1.54, 95% CI 1.36–1.76); evidence for ID without anemia was sparse and non-significant (adjusted OR 0.9, 95% CI 0.6–1.4, from two studies).	Supports the epidemiological basis for including PTB as the second hit; highlights that the ID-without-anemia link remains unresolved.	Meta-analytic/cohort human evidence	Anemia-based definitions may not isolate iron-specific effects; the null ID-without-anemia finding rests on only two studies.
Second hit: Maternal gut ecology and PTB	Miao et al. (2025)	Prospective; 5, 313 Chinese pregnant women, two independent cohorts; early-pregnancy gut metagenomics with functional validation	11 genera and 1 species associated with PTB in early pregnancy; microbial risk scores segregated PTB risk and interacted with host polygenic risk; *C. innocuum* showed 17β-estradiol–degrading activity enriched in PTB	Establishes early-pregnancy maternal gut composition as a predictor of PTB and offers an estrogen-metabolism mechanism, strengthening the maternal-gut arm of the framework	Large, prospective human evidence with mechanistic validation	Single-ancestry (Chinese) cohorts; predictive/associative with functional support, but causal contribution to PTB in humans not established; vaginal/infant transmission not assessed
Relationship between PTB and Maternal transmission	Hiltunen et al. (2022)	Maternal–preterm infant observational cohort comparing spontaneous and iatrogenic PTB	Maternal gut contribution to early neonatal colonization was more pronounced following spontaneous than iatrogenic PTB.	Suggests that the cause and context of PTB may modify maternal–infant microbial sharing.	Human observational evidence	May reflect labor, inflammation, membrane rupture, antibiotics, gestational age, or delivery mode; does not demonstrate transmission of vaginal dysbiosis-associated organisms.

Evidence categories describe directness and design rather than a formal certainty-of-evidence grade. No study has evaluated the complete integrated pathway. Association does not establish mediation or causality.

BV, bacterial vaginosis; CST, community state type; HMO, human milk oligosaccharide; ID, iron deficiency; IDA, iron-deficiency anemia; MCV, mean corpuscular volume; NEC, necrotizing enterocolitis; PTB, preterm birth.

## 3 Maternal vaginal microbiota exposure at birth: transient inoculation and persistent colonization

### 3.1 Mechanisms of vertical microbiota transmission at delivery

Vertical mother-to-infant microbial transmission represents a critical event in early life, but the factors influencing which maternal organisms are acquired and persist in the infant remain incompletely understood (Wang et al., 2020). During vaginal delivery, the infant traverses the birth canal and is exposed to maternal vaginal, fecal, and skin microbiota. A large Dutch birth cohort estimated that 58.5% of infant microbiota composition is attributable to maternal source communities across six maternal niches (Bogaert et al., 2023). However, strain-level metagenomic profiling has revealed that the contributions of these niches are not equivalent in durability as maternal skin and vaginal strains colonize the infant only transiently, whereas maternal gut strains prove more persistent and ecologically better adapted in the infant gut (Ferretti et al., 2018). This finding has been corroborated by another study demonstrating that maternal gut microbiota at 32 weeks of gestation is the primary source of meconium microbiota in vaginally delivered infants, while maternal vaginal microbiota contributes minimally (Xie et al., 2025). By 28 days of life, amplicon sequence variants shared with the maternal rectal microbiota constitute a substantially higher proportion of the neonatal gut than those shared with the maternal vaginal microbiota (Ronde et al., 2025). The distinction between transient exposure and persistent colonization is critical because the vaginal route proposed in the first hit of the framework may operate primarily as a transient immune-priming event at delivery, with any immune-priming effect remaining to be established, whereas the more durable microbiome alterations may arise through gut-derived strains transmitted via the hypothesized entero-mammary pathway or through direct transfer from the maternal gut. The relative contribution of each route remains an active area of investigation, and our framework should be interpreted in this context.

Cesarean delivery (CD) can significantly disrupt this transmission process, though differences may converge by 6–12 months, particularly with breastfeeding (Bogaert et al., 2023). CD-born infants acquire maternal skin and oral microbiota rather than vaginal and fecal microbiota, resulting in delayed colonization by key commensals such as *Bifidobacterium* and premature dominance by opportunistic taxa including *Klebsiella* and *Enterococcus* (Bogaert et al., 2023). Strain inheritance analyses have confirmed that cesarean born infants show a striking lack of maternal strain transmission at birth, with increased relative abundances of non-gut species and delayed transition from an oxygen-tolerant to an oxygen-intolerant microbial community, differences that persist up to two years of life (Korpela et al., 2018; Podlesny and Fricke, 2021). In one study, CD-born neonates exhibited significantly lower abundance of *Escherichia, Bifidobacterium*, and *Bacteroides* compared with vaginally delivered neonates, along with lower serum IgG, reduced IL-12p70, and a significantly reduced IFN-γ/IL-4 ratio, indicating Th1/Th2 imbalance (Lai et al., 2024). Notably, exclusive breastfeeding may partially compensate for this disrupted seeding, suggesting auxiliary routes of mother-to-infant microbial transfer that may help ensure essential microbes and microbial functions are transmitted irrespective of disrupted primary pathways (Bogaert et al., 2023). The Maternal Microbiome Legacy Project which included 585 Canadian mother-infant dyads and used culture-based screening, molecular strain typing, and whole-genome sequencing, detected near-identical vaginal-stool strain sharing in 38.9% of dyads. However, strain sharing occurred irrespective of delivery mode, suggesting that shared strains likely originate from the maternal gut or breast milk rather than from vaginal transmission (Dos Santos et al., 2023). Other studies continue to identify delivery mode as a significant determinant of early neonatal microbiome composition (Bogaert et al., 2023; Lai et al., 2024). Delivery mode therefore functions as both a confounder and a potential effect modifier of the vaginal pathway, while maternal gut and breast milk-related inputs may remain operative after either mode of delivery. This distinction is central to the proposed framework, which considers both transient vaginal exposure and persistent infant colonization while recognizing that their relative contributions remain unresolved.

### 3.2 Vaginal community state types and infant microbial exposure: what is known and unknown

Vaginal microbial communities are commonly classified into community state types (CSTs): CST I (*Lactobacillus crispatus*-dominant), CST II (*L. gasseri-*dominant), CST III (*L. iners*-dominant), CST V (L. jensenii-dominant), and CST IV (diverse anaerobes, low *Lactobacillus*). CST distributions vary substantially by ancestry and socioeconomic contexts. Studies have reported a higher prevalence of CST III and CST IV communities among women of African ancestry and greater *L. crispatus* dominance in some European-ancestry populations (Khan et al., 2024; Powell et al., 2024; Jonouchi et al., 2026). Importantly, race may serve as a proxy for socially driven factors including poverty, education, and vaginal douching practices that differentiate microbiome community structures, as demonstrated in the Pregnancy, Infection, and Nutrition (PIN) Study (Sun et al., 2022).

*L. crispatus-dominant* CST I communities are generally associated with favorable pregnancy outcomes and greater microbiome stability. In the ECHO cohort, CST III (*L. iners-*dominant*)* was associated with an intermediate risk of PTB while CST IV communities have been associated with bacterial vaginosis (BV), PTB, and adverse neonatal outcomes (McKee et al., 2025). Although associations between vaginal CSTs and pregnancy outcomes have been widely studied, considerably less is known about whether CST composition at delivery influences the infant microbiome. A Japanese maternal-neonatal cohort found that higher vaginal *Lactobacillus* abundance in early pregnancy was associated with favorable gestational outcomes, and that bacteria shared between the maternal gut and vaginal microbiome became dominant in the neonatal gut over time (Oguri et al., 2025). Another study found a similar distinction in infant gut microbiome composition between mothers classified as CST III versus CST IV (Bhattacharyya et al., 2023), but the sample (n=25) was too small for definitive conclusions. In a larger cohort of 264 mothers and 266 infants, *L. crispatus* was detected in meconium of vaginally delivered infants within 48 hours of birth; however, the taxonomic resolution of 16S rRNA sequencing could not establish strain-level transmission (Simonyté Sjödin et al., 2025).

A central evidence gap therefore concerns whether maternal vaginal CST at delivery influences the composition, function, or persistence of the infant gut microbiome. Most existing studies have evaluated delivery mode as the principal marker of neonatal microbial exposure, with less attention to the specific vaginal community encountered during birth. Adequately powered longitudinal studies comparing infants born to mothers with CST I, CST III and CST IV communities, using strain-resolved methods and repeated infant sampling, are needed to distinguish transient exposure from persistent colonization.

### 3.3 Potential consequences of dysbiotic vaginal exposure

Under the proposed hypothesis, maternal ID may impair cervicovaginal defenses and thereby increase susceptibility to a low-*Lactobacillus* state or colonization by BV-associated anaerobes such as *Gardnerella, Prevotella*, and *Atopobium*. Exposure to different vaginal communities at birth could plausibly alter early epithelial or immune signaling even if the organisms do not persist in the infant gut (Mann et al., 2024; Bui et al., 2026). Decout et al. (2024) demonstrated that *L. crispatus* surface-layer proteins selectively interact with the anti-inflammatory innate immune receptor DC-SIGN (dendritic cell-specific intercellular adhesion molecule-3-grabbing non-integrin), while *L. iners a*nd *Gardnerella* interact with both pro- and anti-inflammatory receptors. These findings demonstrate species-specific effects on cervicovaginal innate immune signaling and raise the hypothesis that exposure to different vaginal communities could produce different neonatal mucosal signals.

Separately, and independent of a specific immune mechanism, BV during pregnancy has been associated with NICU admission, neonatal sepsis, and respiratory distress even among term births (Dingens et al., 2016). These associations may reflect inflammation, ascending infection, shared maternal risk factors, or other pathways rather than altered microbial transfer at delivery. The proposed vaginal pathway therefore concerns a plausible exposure mechanism, not an established explanation for these neonatal outcomes.

## 4 Maternal iron deficiency as a potential intergenerational microbiome disruptor: the proposed first hit

Having considered the potential consequences of dysbiotic vaginal exposure, we next examine whether maternal ID may contribute to the proposed first hit. Within this framework, the first hit is operationally defined as a potentially measurable alteration in maternal-origin microbial or immunologic inputs that may occur in pregnancies affected by maternal ID, rather than as a single temporally confined event. These inputs may reach the infant through three biologically plausible routes: a vaginal route, in which iron deficiency may alter the cervicovaginal environment and the microbial communities encountered by the infant during birth; a gut-vaginal route, in which iron-deficiency-associated changes in the maternal gut microbiome may indirectly influence vaginal microbial ecology through microbial, metabolic, and immunologic interactions; and a breast milk route, through which maternal iron status may influence postnatal microbial and immunologic inputs involved in infant gut colonization. The breast milk pathway is included as an exploratory extension. These routes are not proposed as independent or equally established mechanisms; rather, they represent maternal-derived, interconnected components of the first hit, supported by varying degrees of direct and indirect evidence. The evidence and biological plausibility underlying each pathway are considered separately below.

### 4.1 Vaginal route: systemic iron deficiency, local iron availability and microbiome interactions

*Lactobacilli*, the predominant bacteria in a healthy vaginal microbiome, use manganese-dependent metabolic pathways that substitute for iron-requiring enzymes, making them uniquely resistant to iron-restricted environments (Bosma et al., 2021). An apparent paradox arises: if *Lactobacillus* requires little or no iron (Elli et al., 2000), how could maternal ID promote its displacement? One possible explanation is that iron deficiency does not affect the vaginal microbiome by starving *Lactobacillus* of iron, but rather by undermining the host defense systems that actively maintain its dominance.

Systemic iron status and iron availability within the cervicovaginal microenvironment represent related but distinct biological compartments. Systemic ID is typically defined by circulating or storage biomarkers such as ferritin, serum iron, transferrin saturation, and hemoglobin (Auerbach, 2023; Cantor et al., 2024). In contrast, vaginal microorganisms interact with iron-containing substrates within the local cervicovaginal environment rather than directly with circulating iron stores; dysbiosis-associated organisms such as *Gardnerella*, for example, acquire iron from iron salts, heme, hemoglobin, and lactoferrin rather than from transferrin, and bacterial taxa differ substantially in their iron-acquisition capacity. Local iron availability may be influenced by host iron-binding proteins such as lactoferrin, cellular iron sequestration governed by hepcidin-ferroportin signaling, inflammation, the presence of heme or hemoglobin, and possibly epithelial turnover (Valenti et al., 2018; Ganz, 2019; Ginzburg, 2023). Hepcidin-driven ferroportin downregulation can sequester iron within genital-tract cells, demonstrating that local genital-tract iron handling can be regulated independently of circulating stores (Izumi et al., 2023).

Consequently, low maternal systemic iron stores should not be assumed to produce proportional reduction in the iron accessible to vaginal microorganisms. In young Burkinabé women, cervicovaginal lactoferrin concentrations tracked genital infection, inflammation, hepcidin, and body iron stores in a complex rather than simple linear fashion (Roberts et al., 2019). Whether systemic iron deficiency alters cervicovaginal iron or heme availability during pregnancy remains unknown and represents an important mechanistic gap.

Against this background, iron deficiency may predispose to vaginal dysbiosis through impaired cervicovaginal mucosal defenses including reduced neutrophil oxidative burst and compromised epithelial barrier integrity (Verstraelen et al., 2005; Valenti et al., 2018; Frost and Drakesmith, 2025). When iron deficiency impairs these host defenses, iron-dependent pathobionts such as *Gardnerella* and *Prevotella* may expand through biofilm formation, protease production, and pH elevation. Experimental evidence indicates that Fe^3+^ enhances *L. jensenii* adhesion to vaginal epithelium, promotes its anti-inflammatory profile, and supports H_2_O_2_-dependent peroxidase activity (Martín et al., 2015). Together, these findings suggest that maternal ID could plausibly influence vaginal community stability, resolving the apparent paradox above, through host defense mechanisms and altered local iron handling. However, whether these mechanisms promote transition toward CST IV communities during pregnancy has not been demonstrated. The primary clinical observation supporting this link comes from a single nested case-control study, in which subclinical iron deficiency at a mean gestational age of 9.2 weeks was a strong independent predictor of BV-like microflora (adjusted OR 4.5, 95% CI 1.4–14.2) (Verstraelen et al., 2005). We emphasize the preliminary nature of this finding as it represents a single unreplicated study and should not be interpreted as conclusive evidence. Prospective longitudinal studies that directly evaluate maternal iron biomarkers, vaginal microbiome composition, and infant outcomes are urgently needed to validate this association.

### 4.2 Gut-vaginal route: a plausible indirect pathway

The second proposed route considers whether ID-associated changes in the maternal gut microbiome may indirectly influence vaginal microbial ecology. Experimental iron-depletion studies in rats have reported enrichment of *Clostridium* and *Enterobacteriaceae*, depletion of butyrate-producing commensals such as *Roseburia* (Dostal et al., 2012; Soriano-Lerma et al., 2022). Persistence of selected microbial alterations following iron repletion has also been reported in experimental models (Coe et al., 2021). However, these findings cannot be directly extrapolated to pregnant women because the magnitude, direction, and reversibility of microbiome changes may depend on the host, dietary context, severity of ID, and route of iron replacement.

Limited human evidence nevertheless suggests that IDA may be associated with differences in selected gut taxa among women of reproductive age. A small study from South India reported lower fecal abundance of the *Lactobacillus acidophilus* group among 8 young women with anemia compared with 26 nonanemic controls, despite similar reported intakes of energy, fiber, iron and milk (Balamurugan et al., 2010). Another study subsequently identified lower *Faecalibacterium* abundance among 15 women with IDA compared with 16 controls and observed an increase in *Faecalibacterium* among participants whose anemia improved following oral iron therapy (Seo et al., 2023). However, the study could not determine whether the increase in *Faecalibacterium* reflected correction of IDA, a direct effect of oral iron, or another time-varying factor. Neither study established a community-wide microbial signature or the direction of the relationship, and both involved small, nonpregnant populations. Furthermore, the classification used by Balamurugan et al. (2010) relied primarily on hemoglobin thresholds, limiting certainty that all observed differences were specifically attributable to IDA. In a longitudinal cohort of 218 pregnant women, maternal anemia was associated with distinct gut microbiota profiles at 6 months postpartum, including *Flavonifractor* and *Enterococcus* with high abundance and *Campylobacter, Coprococcus_2*, and *Desulfovibrio* with low abundance (Mandell et al., 2025). Because anemia was defined using hemoglobin rather than comprehensive iron biomarkers, the relevance of these findings to maternal ID specifically remains uncertain. The same cohort also reported distinct infant gut microbiota profiles at 6 months of age among infants of anemic mothers, with higher abundance of taxa including *Faecalibacterium, Desulfovibrio, and Coprococcus_2* (Mandell et al., 2025).

The gut-vaginal axis provides a plausible route through which these gut perturbations may propagate to the vaginal ecosystem. Proposed mechanisms include shared microbial reservoirs, microbial migration, immune modulation, and metabolite-mediated interactions (Amabebe and Anumba, 2020; Agoni et al., 2026). In a culture-based study of paired vaginal and rectal samples from 132 pregnant women, 28 of the 63 bacterial species identified across the cohort were recovered from both sites. Approximately 36% of participants carried at least one of the same species vaginally and rectally, and identical genotypes were found in 34 of 50 paired isolates of the same species (El Aila et al., 2009). These findings support the rectum as a possible reservoir for vaginal colonization but do not establish the direction, frequency, or temporal dynamics of microbial exchange. A pilot study of 58 pregnant women similarly identified co-residence of BV-associated species in paired rectal and vaginal samples among women with CST IV profiles, supporting the rectum as a possible reservoir (Fudaba et al., 2021). Experimentally, oral administration of *Lactobacillus helveticus* partially restored gut and vaginal microbial diversity following *Gardnerella vaginalis*-induced dysbiosis, providing functional evidence consistent with cross-site ecological interaction (Kim et al., 2026). These observations support biological plausibility but do not establish that ID-induced gut changes cause vaginal dysbiosis in pregnancy.

The gut-vaginal route should therefore be regarded as an indirect and testable component of the first-hit hypothesis rather than an established mechanism. **Figure 1** illustrates the proposed mechanism and the principal points at which direct evidence remains lacking.

**Figure 1:**
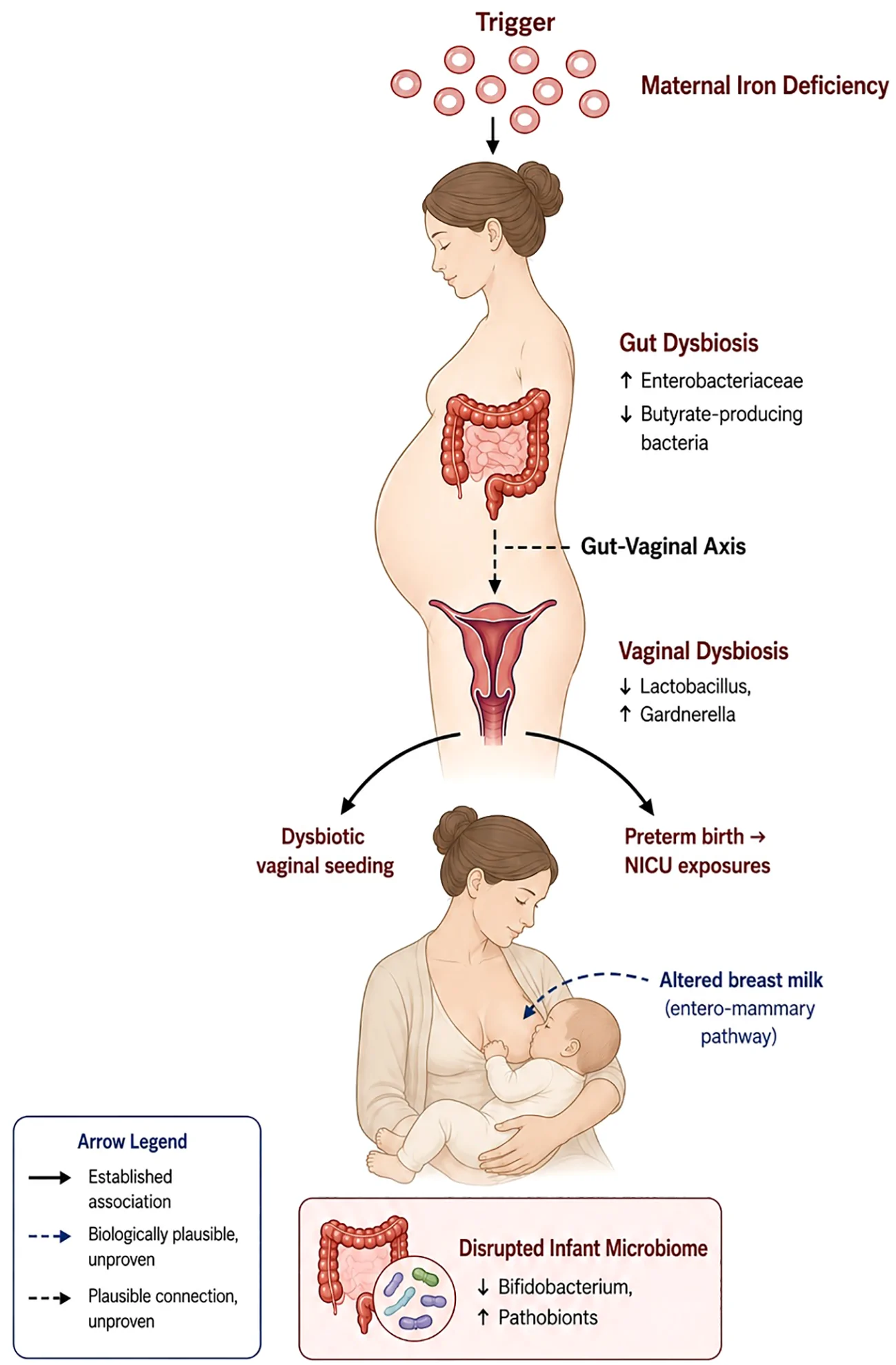
Proposed maternal gut–vaginal pathway and exploratory entero-mammary extension within the first-hit hypothesis. Maternal ID may alter maternal gut microbial ecology, potentially influencing vaginal and breast-milk-derived microbial inputs to the infant. These pathways remain unproven.

### 4.3 Breast milk route: postnatal microbial and immunologic inputs

Human breast milk is an important postnatal route of maternal-to-infant microbial transfer, contributing approximately 28% of infant gut microbiota during the first month of life (Pannaraj et al., 2017). Beyond its microbial content, breast milk also contains cytokines like IL-6, IL-8, IL-10, and tumor necrosis factor-α that provide immune modulation and protection against infections, but two other critical components, lactoferrin and human milk oligosaccharides (HMOs), may shape infant gut colonization (Yi and Kim, 2021).

Breast milk lactoferrin is present at high concentration and plays a dual role through direct antimicrobial activity against pathogens and prebiotic promotion of *Bifidobacterium* and *Lactobacillus* colonization in the infant gut (Yi and Kim, 2021; Kulesza-Brończyk et al., 2023). Mastromarino et al. (2014) demonstrated that neonatal fecal lactoferrin concentration was associated with maternal mature-milk lactoferrin concentration (P = 0.030). Fecal abundances of *Bifidobacterium* and *Lactobacillus* were also associated with fecal lactoferrin at day 3 of life (P = 0.017 and P = 0.026, respectively). These findings support an association between breast milk lactoferrin and early beneficial microbial abundance but do not establish causality.

Whether maternal ID reduces breast milk lactoferrin concentration remains uncertain. A prospective study of 200 mothers (100 anemic, 100 nonanemic) found no correlation between maternal hemoglobin, serum iron, ferritin, or transferrin saturation and breast milk lactoferrin concentration at day 1, 14 weeks, or 6 months (Shashiraj et al., 2006). Similarly, a study conducted in northern Kenya found no evidence that maternal IDA reduced lactoferrin delivery through breast milk, consistent with the possibility that milk lactoferrin is maintained despite variation in maternal iron status (Fujita et al., 2022). In contrast, a recent prospective Chinese cohort of 160 mothers reported an association between prenatal mean corpuscular volume (MCV) and longitudinal lactoferrin trajectories (PMCV×time = 0.011) (Zhou et al., 2025). However, MCV is a nonspecific marker, and this finding requires replication using specific iron biomarkers. Breastfeeding frequency, lactation stage, maternal nutrition, inflammation, and population context may also influence lactoferrin concentrations.

Studies of the breast milk microbiome have commonly detected genera including *Staphylococcus, Streptococcus, Lactobacillus, Pseudomonas, and Bifidobacterium*, although findings vary across populations and sampling and analytical methods (Zimmermann and Curtis, 2020; Yi and Kim, 2021). Established determinants of its composition include maternal BMI, mode of delivery, lactation stage, geographic location, antibiotic exposure, breastfeeding practices (direct vs pumped), and maternal diet particularly fiber, fat, and protein intake (Moossavi et al., 2019; Zimmermann and Curtis, 2020; Cortes-Macías et al., 2021). Although maternal intake of macronutrients, micronutrients, and bioactive compounds may influence milk bacterial composition, results remain inconsistent and methodologically limited (Sindi, 2025). The direct effect of maternal ID on the breast milk microbiome specifically has not been directly examined in a published study. However, there are indirect mechanisms that suggest biological plausibility. One study reported lower IgA, IgG, C3, and C4 in milk from anemic mothers, although anemia was not necessarily attributable exclusively to ID (França et al., 2013). A proposed entero-mammary pathway, an exploratory extension of the first hit, raises the possibility that ID-associated changes in the maternal gut could influence milk microbial composition. However, the existence and quantitative contribution of this route in humans remain uncertain (Sindi, 2025). Moreover, dietary quality and other social or nutritional factors associated with maternal ID could independently influence both maternal and milk microbial communities (França et al., 2013).

HMOs add further complexity to this picture. HMOs selectively support beneficial organisms, particularly *Bifidobacterium infantis*, which possesses a unique enzymatic system for HMO metabolism (Davis et al., 2022; Kiely et al., 2023; Melki and Litvak, 2024). A Finnish population-based cohort (n=517) demonstrated that HMOs exhibit age-dependent and structure-specific associations with infant gut microbiota extending beyond breastfeeding, and that maternal secretor status influences gut microbiota beta diversity (Ovaska et al., 2026). HMO composition is shaped by maternal genetics, BMI, parity, ethnicity, and dietary factors, but its relationship with maternal iron status has not been studied.

Thus, breast milk is an established determinant of infant microbial and immunologic development, but its role in transmitting a maternal ID-associated first hit remains hypothetical. Current evidence does not consistently show that maternal ID alters milk lactoferrin or HMO composition. Prospective studies integrating maternal iron biomarkers with milk lactoferrin, HMO, immune components, microbial profiles, and longitudinal infant stool sampling are needed to test this pathway.

## 5 Preterm birth as an amplifier of disrupted transfer: the proposed second hit

Maternal IDA is associated with an approximately 1.5-fold increase in the odds of PTB (OR 1.54, 95% CI 1.36–1.76) (Benson et al., 2022). By contrast, evidence for ID without anemia is sparse and non-significant (adjusted OR 0.9, 95% CI 0.6–1.4, from only two studies), representing a significant research gap that warrants dedicated investigation (Lawani et al., 2026). Within the proposed framework, when maternal ID or IDA is followed by PTB, prematurity and NICU-associated exposures constitute the second hit and may compound any preceding alterations in maternal-origin microbial or immunologic inputs.

### 5.1 The preterm infant microbiome: a uniquely vulnerable ecosystem

Preterm infants develop a particularly vulnerable intestinal ecosystem characterized by low microbial diversity, depletion of *Bifidobacterium*, and expansion of *Enterobacteriaceae*. These patterns reflect intestinal and immune immaturity combined with frequent exposure to antibiotics, altered feeding, and the NICU environment (Tao et al., 2026). *Proteobacteria*-dominant dysbiosis has been associated with necrotizing enterocolitis (NEC), late-onset sepsis (LOS), and adverse neurodevelopmental outcomes. A large prospective cohort study demonstrated that LOS cases had specific gut microbiome signatures, with higher *Bacilli* abundance and a lack of anaerobic bacteria, before disease onset (Graspeuntner et al., 2019). Lee et al. (2021) confirmed that causative pathogens in neonatal sepsis could be traced to prior gut colonization in 40% of cases.

The vulnerability created by this disrupted microbial ecosystem may be compounded by altered immune development associated with maternal ID. Maternal ID has been linked to impaired neonatal immune development and greater susceptibility to infection during infancy (Ataide et al., 2023). Infants born to iron-deficient mothers may also have reduced iron stores that persist during the first year of life (Davidson et al., 2023; Patel et al., 2025). Because iron contributes to neutrophil oxidative killing, cytokine production, and lymphocyte differentiation (Frost and Drakesmith, 2025), maternal ID could plausibly influence both infant immune competence and microbial succession. Whether these effects amplify prematurity and NICU-associated dysbiosis remains unknown. The framework therefore predicts that preterm infants born to mothers with biomarker-confirmed ID might exhibit greater *Proteobacteria* dominance and more pronounced depletion of immunoregulatory *Bifidobacterium* than preterm infants born to iron-replete mothers. This prediction is directly testable in longitudinal mother–infant studies.

### 5.2 Integrating the two hits

The proposed pathway creates two source-defined and potentially interacting influences on the infant’s early microbial inheritance. Although presented as first and second hits for conceptual clarity, they are not separated by a rigid temporal boundary and may overlap around birth and during the early postnatal period.

#### 5.2.1 First hit: altered maternal-origin inputs

In the proposed model, an infant born vaginally to a mother with ID or an inflammatory, low-*Lactobacillus* vaginal community may encounter a different mixture of microorganisms and microbial products, including greater exposure to *Gardnerella, Prevotella*, and other BV-associated organisms. Whether such exposure influences infant immune development or subsequent *Bifidobacterium* colonization remains unknown. Persistent colonization of the infant gut by vaginally derived organisms has not been established, and cesarean delivery may substantially reduce or bypass this route. Maternal gut-derived strains and breast-milk inputs, however, may remain relevant regardless of delivery mode.

#### 5.2.2 Second hit: PTB and the NICU environment

If PTB occurs in a pregnancy affected by maternal ID, the infant may subsequently encounter exposures associated with prematurity and neonatal intensive care. These include broad-spectrum antibiotics, which can further deplete *Bifidobacterium*; delayed or limited human-milk feeding, which may reduce HMO-mediated prebiotic support; and exposure to nosocomial organisms capable of occupying ecological niches left vacant by depleted commensals (Zwittink et al., 2018; Reyman et al., 2022; Thänert et al., 2024). The central hypothesis is therefore not that the two hits operate as independent pathways, but that prematurity and NICU-associated pressures may amplify any preceding alteration in maternal-origin microbial or immunologic inputs.

Hiltunen et al. (2022) found that spontaneous PTB (vs. iatrogenic) was associated with greater maternal-to-infant gut microbial transmission. This finding, however, may reflect differences in labor, inflammation, membrane rupture, antibiotic exposure, gestational age, or delivery mode. It does not demonstrate maternal-to-infant transmission of vaginal dysbiosis-associated organisms. Accordingly, its relevance to the proposed first hit remains suggestive rather than confirmatory. A prospective study of 5, 313 Chinese pregnant women across two cohorts identified early-pregnancy maternal gut microbial features associated with subsequent PTB. These findings support the relevance of maternal gut microbial ecology to the PTB pathway but do not establish causality or evaluate its relationship with maternal ID or maternal-to-infant microbial transmission (Miao et al., 2025).

Overall, the proposed framework hypothesizes but does not assume that maternal ID causes vaginal dysbiosis or that vaginal dysbiosis mediates the association between ID and PTB. Maternal inflammation, infection, diet, socioeconomic conditions, antibiotic exposure, delivery mode, gestational age, and infant feeding may independently or jointly influence several pathway components. In its fullest hypothesized form, maternal ID may contribute to altered maternal microbial or immunologic inputs, while vaginal inflammation and PTB may modify exposures at delivery. Each connection remains independently testable. **Figure 2** presents the proposed double-hit hypothesis and distinguishes established associations from biologically plausible but unproven relationships.

**Figure 2:**
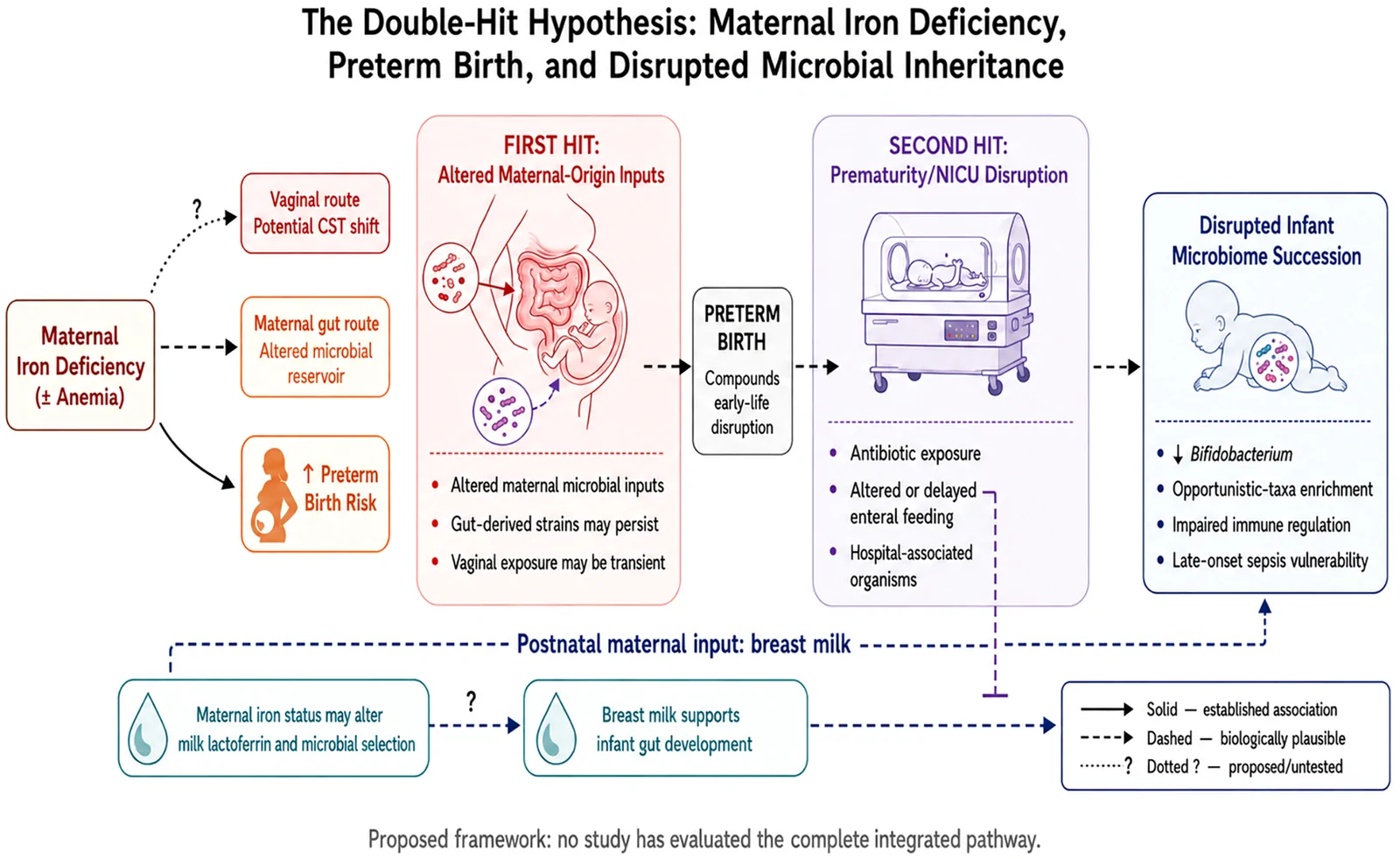
The double-hit hypothesis linking maternal iron deficiency, preterm birth, and disrupted infant microbiome succession. Maternal ID may alter maternal-origin microbial and immunologic inputs through vaginal and maternal gut pathways, constituting the proposed first hit. The breast milk pathway is included as an exploratory extension. If PTB occurs, prematurity-related vulnerability and NICU-associated exposures may constitute a second hit that further disrupts infant microbial succession. Solid arrows indicate established associations, dashed arrows indicate biologically plausible relationships, and dotted arrows indicate proposed or untested relationships. No study has evaluated the complete integrated pathway.

## 6 Translational implications and candidate interventions

The proposed framework identifies potentially modifiable pathways but does not yet provide sufficient evidence to recommend interventions specifically targeting an ID-associated disruption of maternal-to-infant microbial transmission. Translation should therefore proceed sequentially: establishing the proposed relationships, testing their reversibility, evaluating mediation and interaction, and only then examining targeted interventions.

Screening and treatment of maternal ID or IDA, human milk support, and neonatal antibiotic stewardship have clinical rationales independent of this hypothesis. However, their ability to prevent or mitigate the proposed microbiome-related first or second hit remains untested. Candidate microbiome-directed strategies include defined vaginal live biotherapeutics, neonatal probiotics, enteral lactoferrin supplementation, and, in carefully controlled research settings, vaginal seeding after cesarean delivery. These interventions differ substantially in their biological targets, evidence base, and safety considerations and should not be treated as equivalent.

LACTIN-V is an investigational live biotherapeutic (LBP) designed to establish a defined *L. crispatus* strain in the vagina and has reduced BV recurrence following antibiotic treatment in nonpregnant women (Cohen et al., 2020). Its safety and efficacy during pregnancy and its ability to modify maternal-to-infant microbial transfer are still under investigation. By contrast, vaginal seeding exposes an infant to an incompletely defined maternal vaginal community. The latter may transmit pathogenic organisms and could be particularly inappropriate when maternal dysbiosis is present; consequently, ACOG and the AAP do not recommend vaginal seeding outside the context of institutional review board-approved research protocols (Nolt et al., 2022; Wharton and Birsner, 2017).

Neonatal probiotics and enteral lactoferrin supplementation also remain investigational, and their ability to modify infant microbial succession or offset a proposed maternal ID-associated first hit has not been established. Use of these approaches specifically within the proposed framework should therefore be evaluated only after the relevant biological pathways, target population, screening procedures, and safety criteria have been defined.

## 7 Conclusion

This paper proposes a double-hit framework in which maternal ID may alter maternal-origin microbial and immunologic inputs, while PTB and NICU-associated exposures further disrupt infant microbial succession. Although the integrated pathway remains unproven, it provides a biologically plausible and testable explanation for how maternal ID could influence infant microbial and immune development. A single prospective study integrating biomarker-confirmed maternal iron status, serial vaginal CST sampling across gestation, breast milk composition, and longitudinal infant stool sampling through the first year could allow direct testing of whether maternal ID and PTB independently or jointly influence infant microbial succession from birth onward.
